# Carotenoid biosynthetic genes in *Brassica rapa*: comparative genomic analysis, phylogenetic analysis, and expression profiling

**DOI:** 10.1186/s12864-015-1655-5

**Published:** 2015-07-03

**Authors:** Peirong Li, Shujiang Zhang, Shifan Zhang, Fei Li, Hui Zhang, Feng Cheng, Jian Wu, Xiaowu Wang, Rifei Sun

**Affiliations:** Department of Chinese Cabbage, Institute of Vegetables and Flowers, Chinese Academy of Agricultural Sciences, Zhongguancun Nandajie No. 12, Beijing, 100081 P. R. China

**Keywords:** Biosynthetic pathway, Carotenoid biosynthetic genes, Comparative genomics, Expression analysis, *Brassica rapa*

## Abstract

**Background:**

Carotenoids are isoprenoid compounds synthesized by all photosynthetic organisms. Despite much research on carotenoid biosynthesis in the model plant *Arabidopsis thaliana*, there is a lack of information on the carotenoid pathway in *Brassica rapa*. To better understand its carotenoid biosynthetic pathway, we performed a systematic analysis of carotenoid biosynthetic genes at the genome level in *B. rapa*.

**Results:**

We identified 67 carotenoid biosynthetic genes in *B. rapa*, which were orthologs of the 47 carotenoid genes in *A. thaliana*. A high level of synteny was observed for carotenoid biosynthetic genes between *A. thaliana* and *B. rapa*. Out of 47 carotenoid biosynthetic genes in *A. thaliana*, 46 were successfully mapped to the 10 *B. rapa* chromosomes, and most of the genes retained more than one copy in *B. rapa*. The gene expansion was caused by the whole-genome triplication (WGT) event experienced by *Brassica* species. An expression analysis of the carotenoid biosynthetic genes suggested that their expression levels differed in root, stem, leaf, flower, callus, and silique tissues. Additionally, the paralogs of each carotenoid biosynthetic gene, which were generated from the WGT in *B. rapa*, showed significantly different expression levels among tissues, suggesting differentiated functions for these multi-copy genes in the carotenoid pathway.

**Conclusions:**

This first systematic study of carotenoid biosynthetic genes in *B. rapa* provides insights into the carotenoid metabolic mechanisms of *Brassica* crops. In addition, a better understanding of carotenoid biosynthetic genes in *B. rapa* will contribute to the development of conventional and transgenic *B. rapa* cultivars with enriched carotenoid levels in the future.

**Electronic supplementary material:**

The online version of this article (doi:10.1186/s12864-015-1655-5) contains supplementary material, which is available to authorized users.

## Background

Carotenoids represent a group of natural pigments derived from the general isoprenoid biosynthetic pathway. The reaction fundamentals and enzymes involved in the carotenoid pathway have been well studied in *Arabidopsis thaliana* [1]. Carotenoids are mainly synthesized from isopentenyl diphosphate (IPP) and dimethylallyl diphosphate produced by the 2-C-methyl-D-erythritol-4-phosphate (MEP) pathway. The enzymes from the upstream MEP pathway include 1-deoxy-D-xylulose-5-phosphate synthase (DXS), 1-deoxy-D-xylulose-5-phosphate reductoisomerase (DXR), 2-C-methyl-D-erythritol 4-phosphate cytidylyltransferase, 4-diphosphocytidyl-2-C-methyl-D-erythritol kinase (CMK), 2-C-methyl-D-erythritol 2,4-cyclodiphosphate synthase (MDS), 4-hydroxy-3-methylbut-2-enyl diphosphate synthase (HDS), and 4-hydroxy-3-methylbut-2-enyl diphosphate reductase (HDR).

Additionally, isopentenyl pyrophosphate isomerase (IPPI) catalyzes the isomerization of dimethylallyl diphosphate to IPP, while geranyl pyrophosphate synthase (GPS) transforms IPP to geranyl pyrophosphate (GPP) [2–12]. The first key step for carotenoid biosynthesis is the production of a 40-carbon phytoene from paired geranylgeranyl pyrophosphate (GGPP) molecules. The process is catalyzed by enzyme phytoene synthase (PSY) [13, 14]. Then, a series of desaturases and isomerases participate in the generation of lycopene (colored) from phytoene (no color), including phytoene desaturase (PDS), ζ-carotene desaturase (ZDS), 15-*cis*-ζ-carotene isomerase (Z-ISO), and carotenoid isomerase (CRTISO) [15–18]. Lycopene represents the first branch point of the carotenoid pathway and is catalyzed by two competing enzymes, lycopene β-cyclase and lycopene ε-cyclase, which result in the production of β-carotene and α-carotene, respectively [19]. Then, carotenoid cleavage dioxygenases (CCD) participate in the formation of apocarotenoid [20]. Cyclic carotenes are further modified by two different types of carotenoid hydroxylases in *A. thaliana*: non-heme di-iron enzymes (BCH type) and cytochrome P450 enzymes (CYP97 type), which include BCH1, BCH2, LUT5, LUT1, and CYP97B3 [21–23]. Zeaxanthin then enters the xanthophyll cycle through the stepwise activities of zeaxanthin epoxidase (ZEP) and violaxanthin de-epoxidase (VDE) [24, 25]. The pigments antheraxanthin and violaxanthin produced by the above processes are further converted to neoxanthin by neoxanthin synthase (NSY) [24]. Finally, the enzymes 9-cis-epoxycarotenoid dioxygenase (NCED), xanthoxin dehydrogenase (ABA2), and abscisic-aldehyde oxidase (AAO3) catalyze violaxanthin and neoxanthin to produce xanthoxin and abscisic acid, respectively [26–31].

The genus *Brassica* includes many vegetable crops, such as *B. rapa*, *Brassica oleracea*, *Brassica napus*, *Brassica parachinensis*, and *Brassica juncea. B. rapa* L. ssp. *pekinensis* (Chinese Cabbage) evolved in China and is an important vegetable crop in Asia. The inner leaves of several cultivars of heading *B. rapa* are orange and yellow, and are abundant in lutein, β-carotene, and prolycopene [32–34]. Because of the previous absence of genome information, little is known about the genes involved in the carotenoid biosynthetic pathway of *B. rapa* [35, 36]. The sequencing and release of the *B. rapa* genome [37], provides a good opportunity to systematically study the carotenoid biosynthetic genes in *B. rapa*. A complete understanding of the carotenogenesis genes is important for elucidating the mechanisms of carotenoid biosynthesis in *B. rapa*, as well as for the breeding of new *B. rapa* varieties with rich carotenoids, which are good for human health.

Whole-genome duplication events provide abundant amounts of genetic material for gene family expansion or the evolution of new genes in plants [38, 39]. *B. rapa* experienced a whole-genome triplication (WGT) event after its divergence from *A. thaliana* and has three subgenomes in its nucleus [40]. The level of gene loss among the three subgenomes of *B. rapa* is biased: fragments with the highest gene densities comprise subgenome LF, fragments with moderate gene densities comprise subgenome MF1, and those with the least genes comprise subgenome MF2 [41, 42]. The fragments of the three subgenomes in *B. rapa* have been well distinguished; therefore, differences in gene loss among these subgenomes can be identified unambiguously. Although the influence of WGT on some gene families has been studied [43, 44], there is presently no detailed information on the evolution of carotenoid biosynthetic genes after the WGT in *B. rapa*. To obtain comprehensive information on the carotenoid biosynthetic pathway in *B. rapa* and to explore the effect of the WGT on these genes, we performed a comparative genomic analysis between *B. rapa* and *A. thaliana* using the genome sequences and annotation information of the two species [37]. We investigated the evolution and functions of carotenoid biosynthetic genes in *B. rapa* by constructing phylogenic trees and analyzing their transcription patterns. The systematic analysis of carotenoid biosynthesis genes in *B. rapa* will improve our understanding of the genetic mechanisms of carotenoid biosynthesis and carotenoid accumulation in *B. rapa* crops.

## Results and discussion

### Identification of carotenoid biosynthetic genes in *B. rapa*

Using the carotenoid biosynthetic enzymes as queries we searched the TAIR and KEGG pathway databases. In *A. thaliana*, 47 potential carotenoid biosynthetic genes were investigated, including 21 genes that participated in the MEP pathway upstream of GGPP and 26 genes encoding carotenoid biosynthetic enzymes. Based on a combination of syntenic and non-syntenic orthology analyses, 67 *B. rapa* carotenoid biosynthetic genes were identified, representing orthologs of 42 out of the 47 *A. thaliana* carotenoid biosynthetic genes (Table 1; Additional file 1: Table S1). The other five *A. thaliana* carotenoid biosynthetic genes (*GGPS6*, *GGPS9*, *GGPS11*, *GGPS12* and *LUT1*) showed no *B. rapa* orthologs. Each carotenoid biosynthetic gene in *B. rapa* was assigned a name based on the enzymatic reaction, similar to those given in the *A. thaliana* carotenoid biosynthetic pathway [45].

**Table 1:** Carotenoid biosynthetic genes identified in Brassica rapa

		*B. rapa*			
Enzyme	*A. thaliana*	Syntenic orthologs			Non-syntenic orthologs
		LF	MF1	MF2	
MEP pathway to GGPP genes					
DXS	*AT4G15560*	*BrDXS1* (Bra033495)	*BrDXS2* (Bra012779)	-	-
DXR	*AT5G62790*	*BrDXR1* (Bra010123)	*-*	*BrDXR2* (Bra035881)	-
MCT	*AT2G02500*	*-*	*BrMCT* (Bra026591)	*-*	-
CMK	*AT2G26930*	*BrCMK* (Bra012040)	*-*	*-*	-
MDS	*AT1G63970*	*-*	*-*	*BrMDS1* (Bra027672)	*BrMDS2* (Bra027770)
HDS	*AT5G60600*	*BrHDS* (Bra002468)	*-*	*-*	*-*
HDR	*AT4G34350*	*BrHDR1* (Bra011522)	*-*	*BrHDR2* (Bra034620)	*-*
IPPI1	*AT5G16440*	*-*	*BrIPPI1* (Bra006354)	*-*	*-*
IPPI2	*AT3G02780*	*BrIPPI2.1* (Bra040599)	*BrIPPI2.2* (Bra021411)	*BrIPPI2.3* (Bra001063)	*-*
GGPS1	*AT4G36810*	*BrGGPS1.1* (Bra011709)	*BrGGPS1.2* (Bra017785)	*BrGGPS1.3* (Bra010576)	*BrGGPS1.4* (Bra028096)
GGPS2 (GGPS5)	*AT2G23800*	*BrGGPS2.1* (Bra039216)	*BrGGPS2.2* (Bra032140)	*-*	
GGPS3	*AT3G14550*	*BrGGPS3.1* (Bra027330)	*BrGGPS3.2* (Bra021562)	*BrGGPS3.3* (Bra001556)	
GGPS7	*AT2G18620*	*-*	*BrGGPS4* (Bra038544)	*-*	
GGPS8	*AT3G14510*	*-*	*BrGGPS8.1* (Bra021565)	*-*	
GGPS11	*AT3G29430*	*-*	*-*	*-*	
GGPS4	*AT2G18640*	*-*	*BrGGPS4* (Bra038544)	*-*	*-*
GGPS6	*AT1G49530*	*-*	*-*	*-*	*-*
GGPS9	*AT3G14530*	*-*	*-*	*-*	*-*
GGPS10	*AT3G20160*	*BrGGPS10.1* (Bra035808)	*-*	*BrGGPS10.2* (Bra001777)	*-*
GGPS12	*AT3G32040*	*-*	*-*	*-*	*-*
GGR	*AT4G38460*	*BrGGR* (Bra011898)	*-*	*-*	*-*
Carotenoid biosynthetic genes					
PSY	*AT5G17230*	*BrPSY1* (Bra008569)	*BrPSY2* (Bra006391)	*BrPSY3* (Bra023603)	*-*
PDS3	*AT4G14210*	*-*	*BrPDS3.1* (Bra032770)	*BrPDS3.2* (Bra010751)	*-*
Z-ISO	*AT1G10830*	*BrZ-ISO* (Bra019899)	*-*	*-*	*-*
ZDS	*AT3G04870*	*-*	*BrZDS* (Bra040411)	*-*	*-*
CRTISO	*AT1G06820*	*-*	*-*	*BrCRTISO* (Bra031539)	*-*
CRTISO2	*AT1G57770*	*-*	*-*	*BrCRTISO2* (Bra027908)	*-*
LYC	*AT3G10230*	*BrLYC* (Bra029825)	*-*	-	*-*
LUT2	*AT5G57030*	*BrLUT2.1* (Bra002769)	*BrLUT2.2* (Bra006838)	-	*BrLUT2.3* (Bra020718)
CHY1	*AT4G25700*	*BrCHY1.1* (Bra013912)	*BrCHY1.2* (Bra019145)	-	-
CHY2	*AT5G52570*	*BrCHY2.1* (Bra003121)	-	-	-
LUT5	*AT1G31800*	-	*BrLUT5* (Bra038437)	-	-
CYP97B3	*AT4G15110*	-	*-*	*BrCYP97B3* (Bra038092)	-
LUT1	*AT3G53130*	-	*-*	*-*	-
ZEP	*AT5G67030*	*BrZEP1* (Bra012127)	*-*	*BrZEP2* (Bra037130)	-
VDE	*AT1G08550*	*BrVDE* (Bra018616)	*-*	*-*	-
NSY	*AT1G67080*	-	*BrNSY* (Bra034026)	*-*	-
CCD7	*AT2G44990*	*-*	*BrCCD7* (Bra040330)	*-*	-
CCD8	*AT4G32810*	*BrCCD8* (Bra011384)	*-*	*-*	-
NCED2	*AT4G18350*	*BrNCED2.1* (Bra013298)	*BrNCED2.2* (Bra012603)	*-*	-
NCED3	*AT3G14440*	*BrNCED3.1* (Bra027336)	*BrNCED3.2* (Bra021558)	*BrNCED3.3* (Bra001552)	-
NCED4	*AT4G19170*	*BrNCED4.1* (Bra013378)	-	*BrNCED4.2* (Bra020970)	-
NCED5	*AT1G30100*	*BrNCED5* (Bra032359)	-	-	-
NCED6	*AT3G24220*	*BrNCED6* (Bra015002)	-	-	-
NCED9	*AT1G78390*	*BrNCED9.1* (Bra035033)	*BrNCED9.2* (Bra008358)	-	-
ABA2	*AT1G52340*	*BrABA2.1* (Bra018964)	*BrABA2.2* (Bra014323)	-	-
AAO3	*AT2G27150*	-	*BrAAO3* (Bra034325)	-	-

**Fig. 1:**
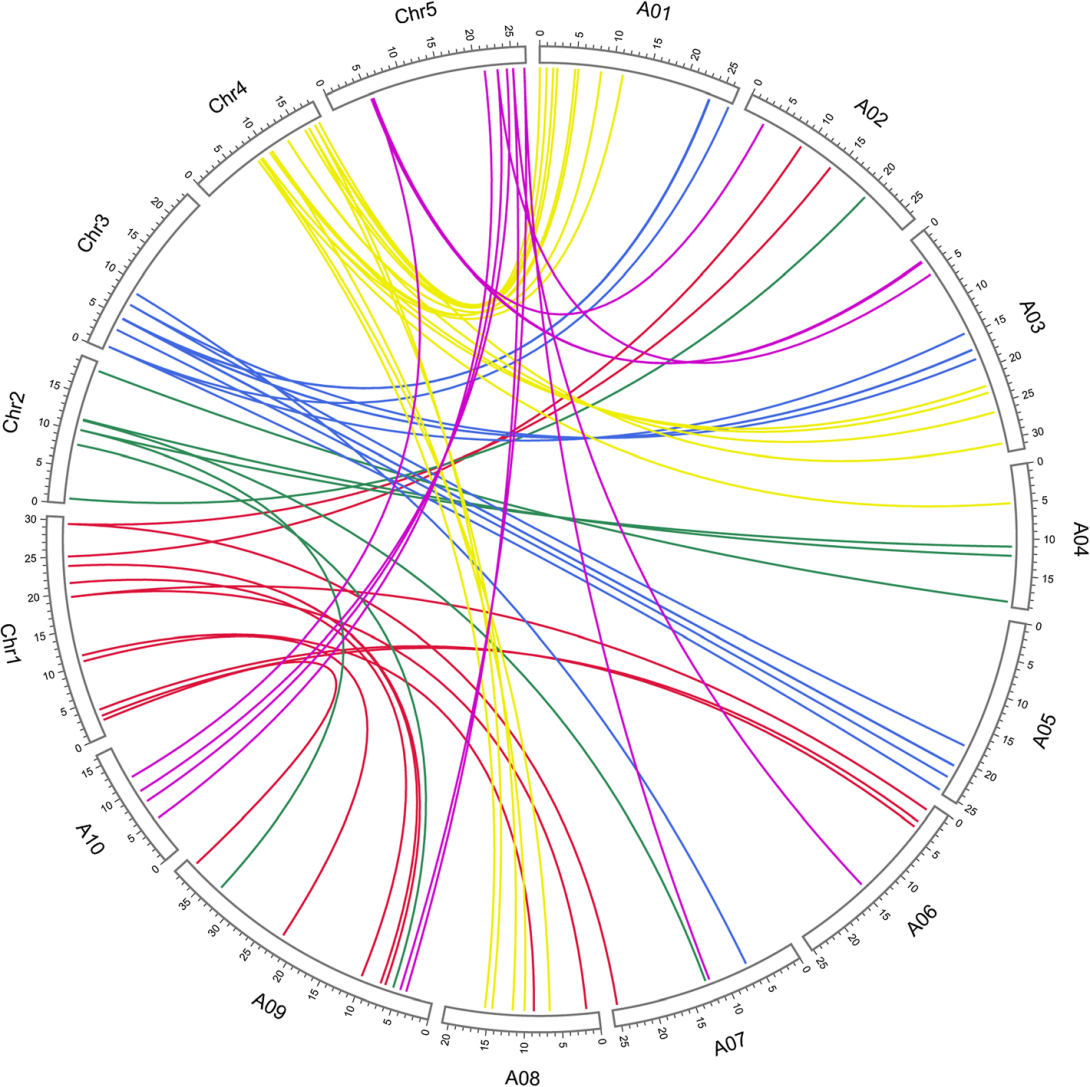
Ideogram of syntenic carotenoid biosynthetic genes in *Brassica rapa* and *Arabidopsis thaliana*. Ch1 to Ch5 are *A. thaliana* chromosomes, and A01 to A10 are *B. rapa* chromosomes. All ranges are to scale

Among the 67 carotenoid biosynthetic genes in *B. rapa*, 64 were syntenic orthologs of the 40 *A. thaliana* carotenoid biosynthetic genes (Fig. 1), and only three *B. rapa* carotenoid biosynthetic genes had no syntenic relationships. The carotenoid biosynthetic genes have expanded in the genome of *B. rapa*. The multiple copies of the carotenoid biosynthetic genes in *B. rapa* that are syntenic to genes in *A. thaliana* were generated from the WGT. In addition, 37 of the 42 *A. thaliana* carotenoid biosynthetic genes had less than three syntenic orthologs in *B. rapa* as a result of gene fractionation following the triplication event. GGPS is encoded by a multigene family with 12 members in *A. thaliana* [10], but *GGPS6*, *GGPS9*, *GGPS11* and *GGPS12* orthologs were not found in *B. rapa. GGPS4* and *GGPS7* form a tandem array in *A. thaliana* and correspond to one gene (Bra038544) in *B. rapa*. Furthermore, *GGPS1*, *GGPS2*, *GGPS3*, *GGPS7*, *GGPS8*, and *GGPS11* share the same non-syntenic ortholog (Bra028096) in *B. rapa*.

### Chromosomal and subgenomic distributions

**Fig. 2:**
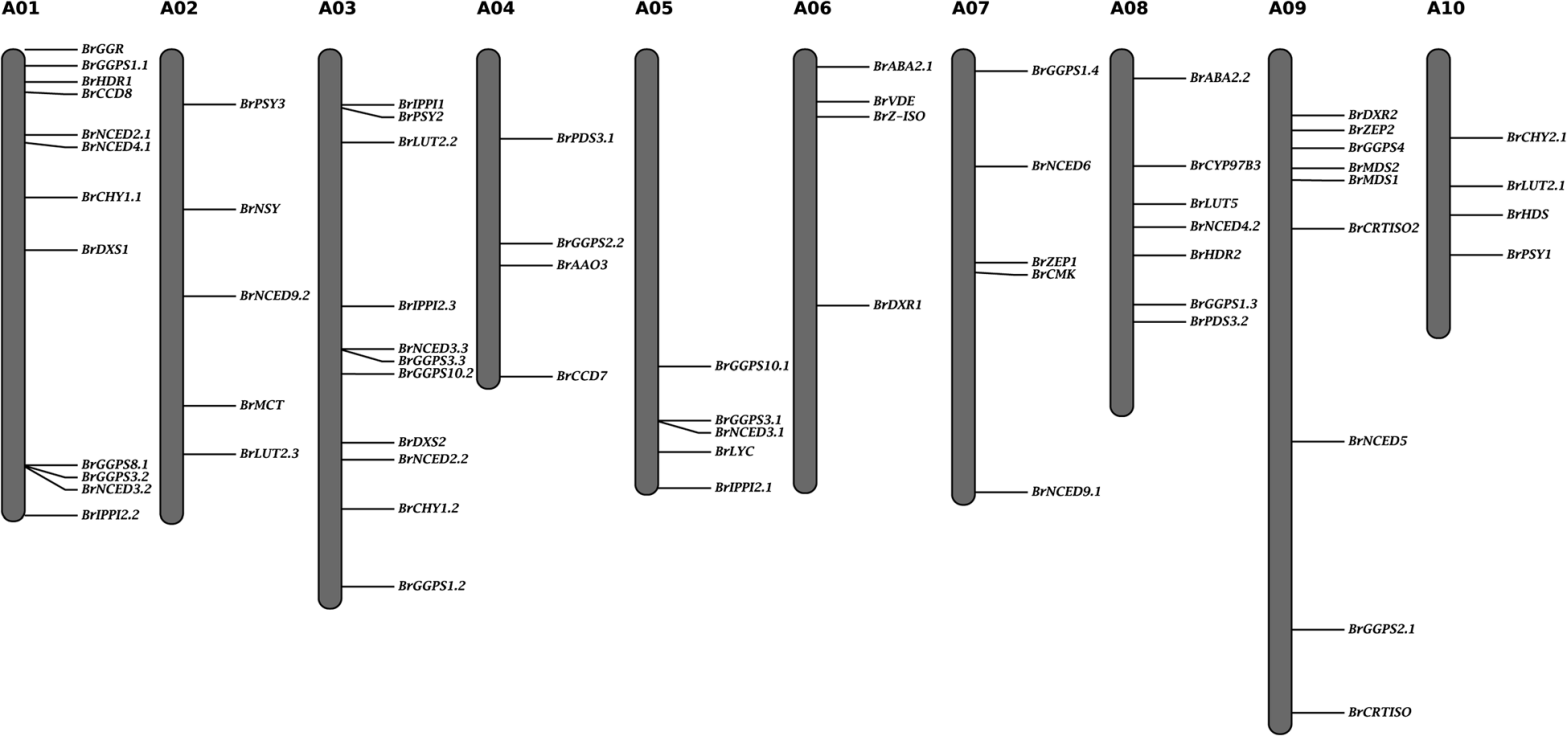
Genomic distribution of 66 carotenoid biosynthetic genes on the 10 chromosomes of *Brassica rapa*

A diagrammatic representation of the chromosomal distribution of carotenoid biosynthetic genes on the 10 *B. rapa* chromosomes is depicted in Fig. 2. In total, 66 of 67 *B. rapa* carotenoid biosynthetic genes were mapped to the 10 chromosomes, with 12, 5, 11, 4, 5, 4, 5, 7, 9, and 4 *B. rapa* carotenoid biosynthetic genes being located on chromosomes A01–A10 in the *B. rapa* genome V1.5, respectively (Fig. 2). The remaining gene, *BrZDS* (Bra040411), was assigned to Scaffold000203, which has not yet been assembled to any chromosome according to the *B. rapa* genome V1.5 (Additional file 1: Table S1). Interestingly, there are no tandem duplicated carotenoid genes in *B. rapa.*

The genome structure of *B. rapa* was shaped by the WGT event followed by extensive diploidization [46]. In the genome, fragments with the highest gene densities are in subgenome LF, fragments with moderate gene densities are in subgenome MF1, and the fragments with the least genes are in subgenome MF2 [37, 42]. With this subgenomic information, we then assigned all *B. rapa* carotenoid biosynthesis genes to the three subgenomes. There were 28, 23, and 16 genes located in LF, MF1, and MF2, respectively. There were more genes located in LF, while fewer genes were distributed in MF1 and even fewer genes in MF2. Of the 64 syntenic orthologs, 27 were in LF, 22 were in MF1, and 15 were in MF2. The proportion of total subgenomic *B. rapa* genes was used as the background to calculate the *P*-value using Fisher’s t-test. The *P*-value was 0.715 (>0.05), indicating that the proportion of carotenoid biosynthetic genes in each subgenome of *B. rapa* was not significantly different from the background. These results show that the distribution of carotenoid biosynthetic genes is consistent with the gene fractionation status at the whole-genome level [37, 42]. Based on the determination of these carotenoid biosynthetic genes, the carotenoid biosynthetic pathway in *B. rapa* was thus established.

### Evolution of the *PSY* genes in *B. rapa*

**Fig. 3:**
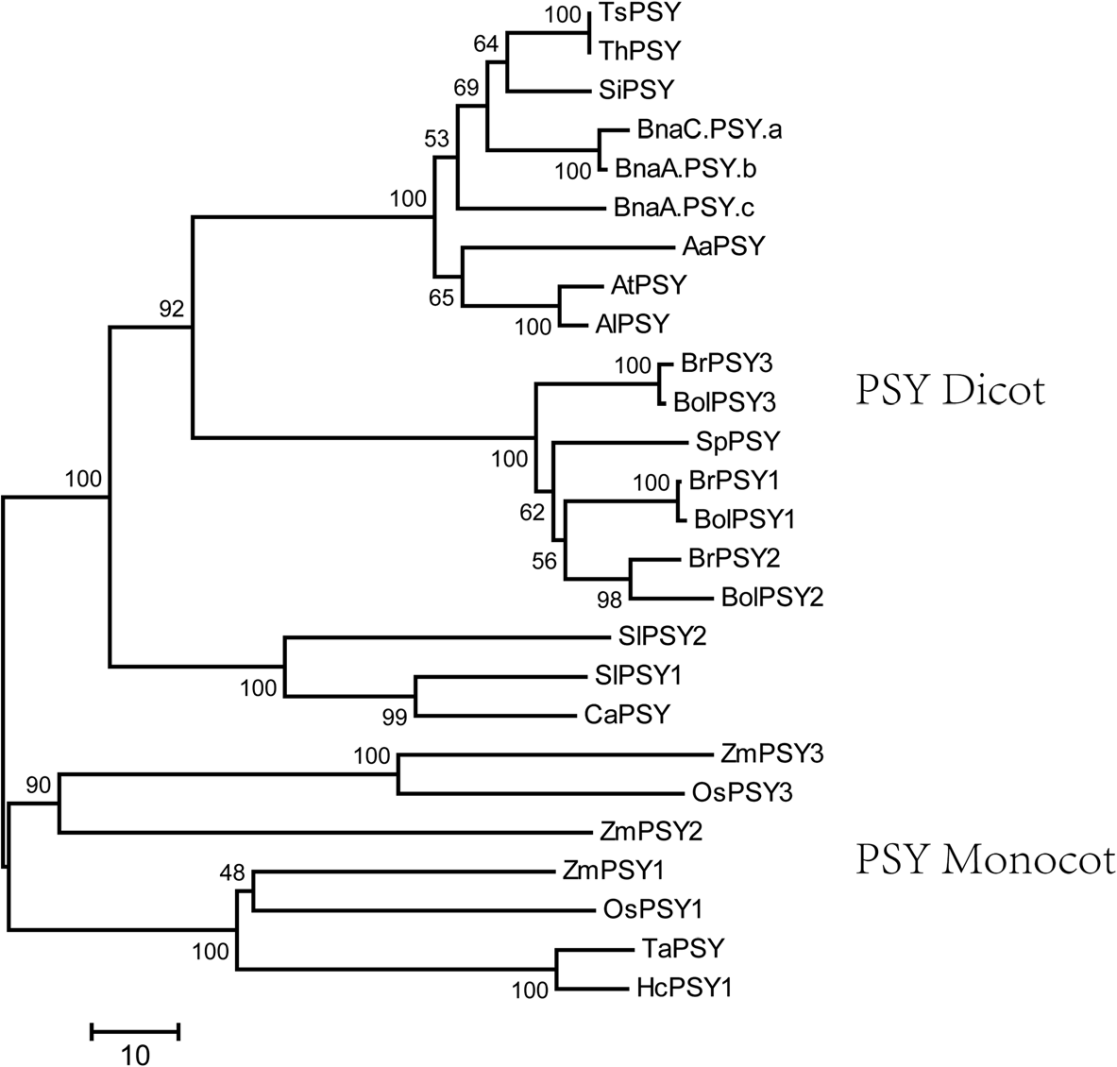
Phylogenetic relationship of the *PSY* gene between *Brassica rapa* and other species. The unrooted tree was generated using the Muscle program by the neighbor-joining method. Bootstrap values from 1000 replicates are indicated at each node. *Ts*: *Thellungiella salsuginea; Th*: *Thellungiella halophila; Si*: *Sisymbrium irio At: Arabidopsis thaliana; Al: Arabidopsis lyrata; Aa*: *Aethionema arabicum Br: Brassica rapa; Bol: Brassica oleracea; Sp: Schrenkiella parvula; Bna: Brassica napus; Sl: Solanum lycopersicum; Ca: Capsicum annuum; Os: Oryza sativa; Zm: Zea mays; Ta: Triticum aestivum;* and *Hc: Hordeum chilense*

The WGT event in the *B. rapa* genome provides a model for the study of the evolutionary fate of multi-copy genes and the effects of polyploidy in economically important crops. To investigate the evolutionary relationship of carotenoid biosynthetic genes in *B. rapa*, separate neighbor-joining trees were generated for the enzyme PSY by aligning the protein sequence with the corresponding orthologs in *Arabidopsis* and other plant species (Fig. 3). The phylogenetic analysis of *PSY* in *B. rapa*, *Arabidopsis*, and other monocot and dicot plant species revealed that *PSY*s cluster into two separate monocot- and dicot-specific clades, where most of the members show a monophyletic pattern of origin. In the Brassicaceae family, the species were separated into two specific clades. As shown in Fig. 3, *PSY1*, *PSY2*, and *PSY3* sequences from *B. rapa* each clustered into groups with their respective *B. oleracea* orthologs on a separate branch. Interestingly, *B. rapa*, *B. oleracea*, and *Schrenkiella parvula* are clustered on one branch, which indicates that the *Brassica* are closer to *S. parvula* than to *Arabidopsis* and that the Brassiceae triplication event occurred near the time of the divergence between Brassiceae and Schrenkiella [46]. However, three *B. napus PSY*s were clustered on one specific branch with the *Arabidopsis* and *Thellungiella PSY*s.

PSY is a rate-limiting enzyme in *Solanum lycopersicum* fruit, *B. napus* seeds, *Gentiana scabra* Bunge flowers, and other plants [47–50], and is a key committed enzyme in the carotenoid biosynthetic pathway [51]. In *A. thaliana*, a single *PSY* gene regulates phytoene synthesis in all tissues [14], while three genes encoding PSY enzymes were retained in *B. rapa* and *B. oleracea* after the WGT event. The *PSY* gene family expansion preceded the speciation of *B. rapa* and *B. oleracea*, dating back to the WGT event [52]. Furthermore, six *PSY*s were retained in *B. napus*; however, we selected only three *B. napus PSY* genes that had complete sequences online. The evolution of carotenoid biosynthetic genes, such as *PSYs*, was consistent with the evolution of *B. rapa*.

### Expression profiles of carotenoid biosynthetic genes in six organs of *B. rapa*

**Fig. 4:**
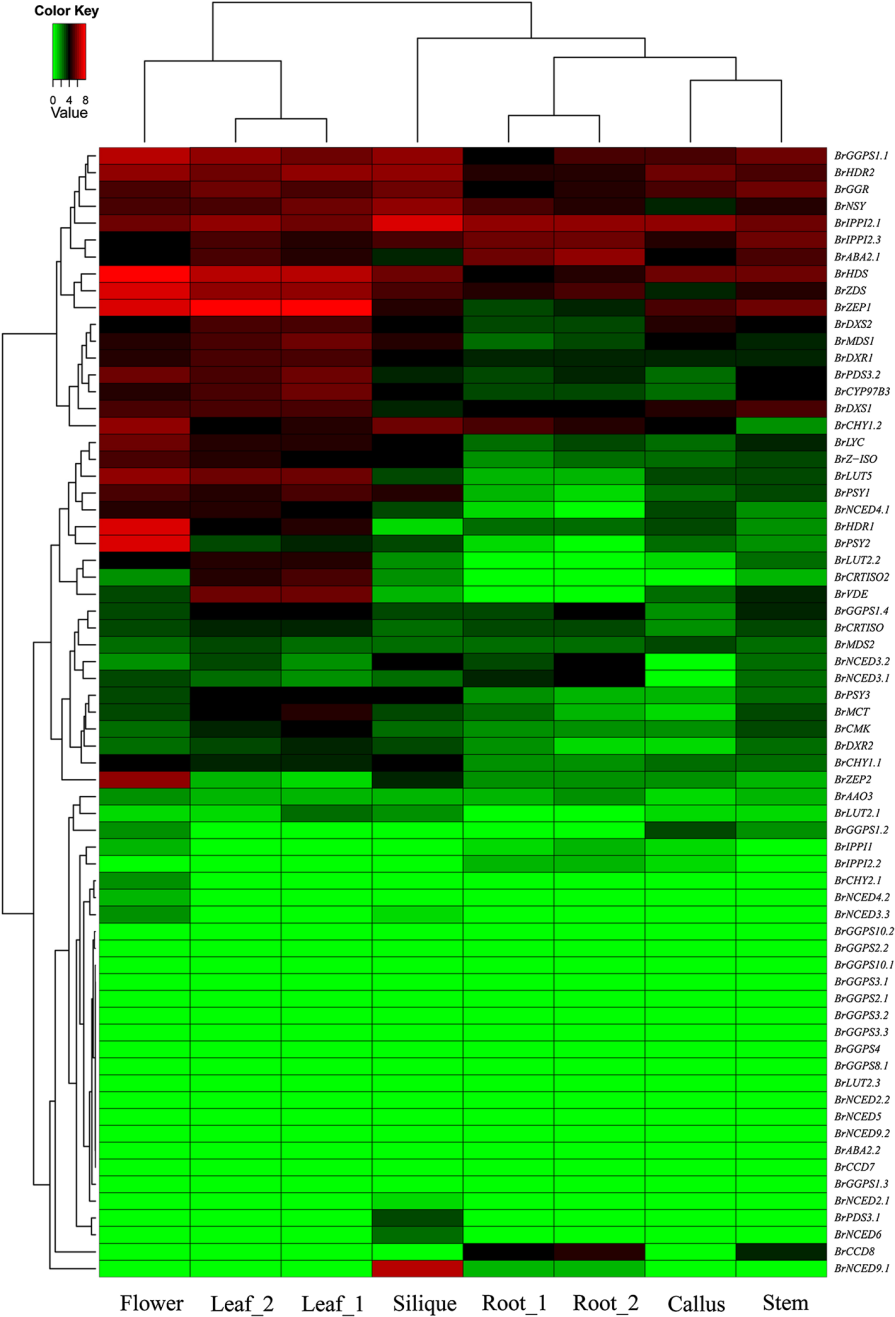
Expression profiles of *Brassica rapa* carotenoid biosynthetic genes in six organs. Transcriptome sequencing was employed to investigate expression patterns of *B. rapa* carotenoid biosynthetic genes. The color scale shown at the top represents FPKM-normalized log2-transformed counts. Green indicates low expression levels, while red indicates high levels

To determine the expression patterns of carotenoid biosynthetic genes in different organs and elucidate their roles in the carotenoid biosynthetic pathway, we performed next-generation transcriptome sequencing (RNA-Seq) [53], as well as a hierarchical-clustering analysis of carotenoid biosynthetic gene expression patterns using the R software [54]. As shown in Fig. 4, the expression levels of the carotenoid biosynthetic genes in the six organs were diverse and could be divided into two groups. Flowers and leaves were in group I, and the flowers had the highest overall expression level and the maximum number of expressed genes among the six tissues. Siliques, roots, stems, and calli were in group II, and calli had the lowest overall expression level, although it was only slightly lower than in the roots.

The *B. rapa* carotenoid biosynthetic genes could be divided into two clusters based on their expression patterns (Fig. 4; Additional file 2: Table S2). Cluster 1 was composed of genes that were expressed in all six organs, while Cluster 2 contained genes that had low expression levels and some organ-specific expression patterns. Cluster 1 included two expression groups. The first group showed a high expression level in all six organs. The second group exhibited a higher expression level in flowers, leaves, and siliques, indicating their roles in the carotenoid biosynthetic pathway in these tissues, than in roots, calli, and stems. The genes belonging to Cluster 2 had lower expression levels and could be subdivided into three groups. The first group was composed of 10 genes that had low expression levels in siliques, roots, calli, and stems. The genes in the second group exhibited low but stable expression levels in all six organs, except *BrZEP2*, which was highly expressed in flowers. This result was consistent with a previous study and indicates that *BrZEP2* plays an important role in the synthesis and accumulation of carotenoids in *B. rapa* flowers [55]. Most genes belonging to the third group exhibited low or undetectable expression levels in all six organs. Interestingly, the gene *BrCCD8* was expressed in stems and roots but could not be detected in flowers, leaves, siliques, or calli, and *BrNCED9.1* was highly expressed in siliques.

According to the expression analysis results, most *B. rapa* carotenoid biosynthetic genes appeared to have similar roles to their orthologs in other species. For example, it has been reported that the *PSY*, *ZDS*, *PDS*, and *ZEP* genes play crucial roles in carotenoid biosynthesis in *A. thaliana* [1]. In *B. rapa*, these genes exhibited either predominant or specific expression patterns in leaves, such as *BrZEP1*, which was highly expressed in flowers and leaves. *A. thaliana* contains a family of 12 genes that are similar to *GGPS*, but only five *GGPS* genes have been shown to be expressed in different tissues during plant development [10]. Although there are 13 duplicated *BrGGPS* genes in *B. rapa*, only *BrGGPS1.1* was highly expressed in the six organs we examined. Moreover, most of the *BrGGPS* genes did not have detectable expression levels, which may be due to functional divergence after the triplication event or because they are specifically expressed at other developmental stages.

### Expression variance among duplicated carotenoid biosynthetic genes in *B. rapa*

**Fig. 5:**
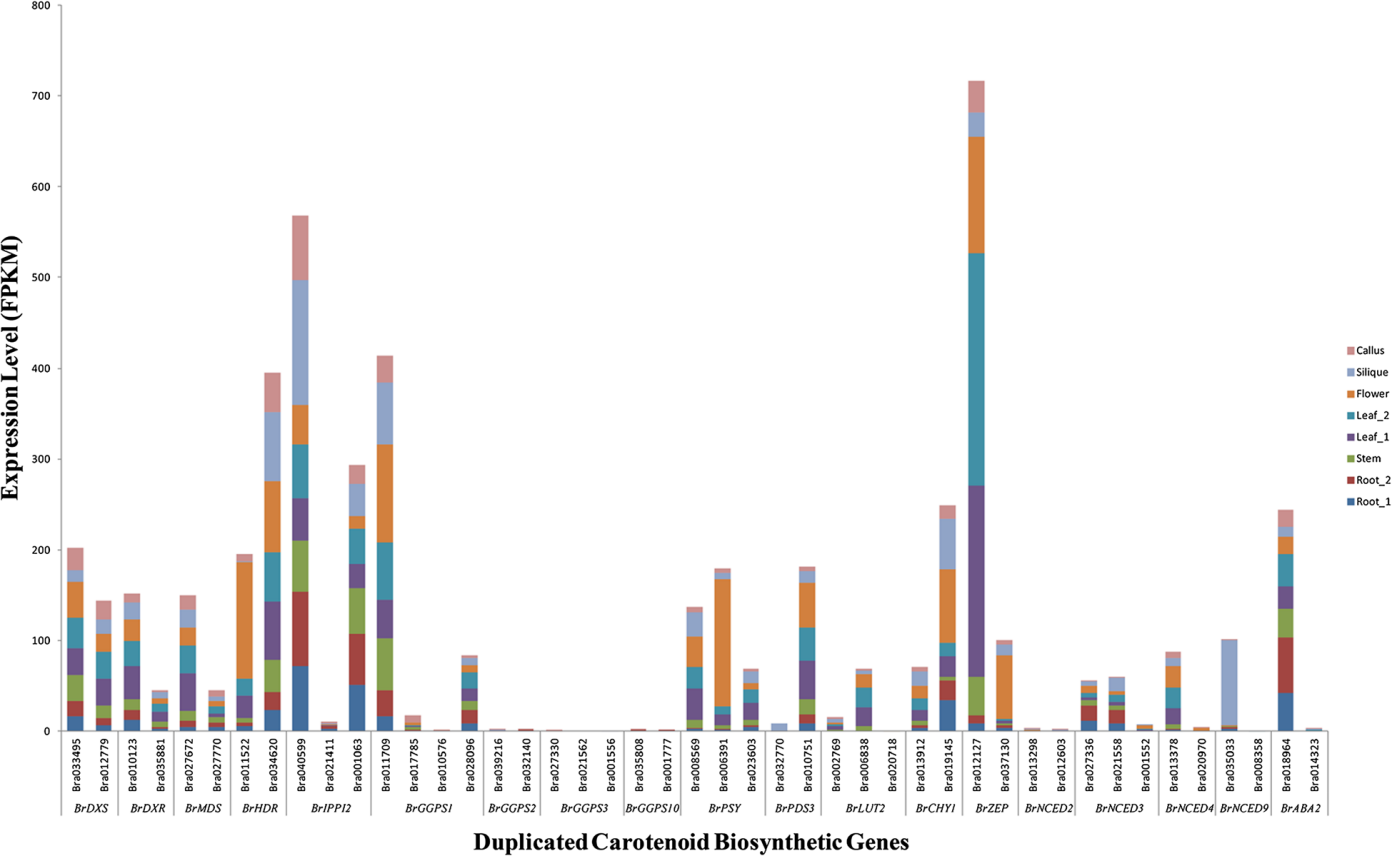
Different transcript levels of duplicated carotenoid biosynthetic genes in *Brassica rapa*

Expression differentiation, which is considered the first step in the functional divergence between duplicated genes, has long been a subject of great interest to geneticists and evolutionary biologists [56–58], because it increases the probability of the retention of duplicated genes in a genome [57]. The duplicated genes that we have described here are paralogous carotenoid biosynthetic genes that originated due to the WGT event in *B. rapa*. The carotenoid biosynthetic genes that have more than one copy were selected to analyze expression variance in *B. rapa*. Analysis of RNA-Seq data generated from *B. rapa* root, leaf, flower, silique, callus and stem tissues suggests that WGT paralogous gene pairs show significant expression differentiation (Fig. 5; Additional file 2: Table S2). In each duplicated carotenoid biosynthetic gene pair, the expression levels change significantly. For example, in some carotenoid biosynthetic genes, such as *BrDXR*, *BrMDS*, *BrHDR*, *BrIPPI2*, *BrGGPS1*, *BrPSY*, *BrPDS3*, *BrLUT2*, *BrCHY1*, *BrZEP*, *BrNCED3*, *BrNCED4*, *BrNCED9*, and *BrABA2*, one copy is much more highly expressed than the other. In other genes, such as *BrDXS*, *BrGGPS2*, *BrGGPS3*, *BrGGPS10*, and *BrNCED2*, the differential expression levels between the copies were not significant. Additionally, the expression levels of each copy of *BrGGPS2*, *BrGGPS3*, and *BrGGPS10* were lower*.* In general, the expression levels of genes in the LF subgenome were significantly higher than the corresponding syntenic genes in the MF1 and MF2 subgenomes (Additional file 3: Figure S1).

The expression differentiation between duplicate genes has been reported in many studies [59–62], and the functional divergence of *PSY* gene expression facilitates the accumulation of high levels of carotenoids in chromoplast-rich tissues in *B. napus* [52]. In this paper, we studied the expression variance of genes that were created during a WGT event using RNA-Seq data. A gene expression analysis revealed an extensive variance between paralogs of each carotenoid biosynthetic gene. These duplicated gene expression variations may be signs of subfunctionalization among different tissues and contribute to an increased complexity in the regulatory networks of the carotenoid pathway after polyploidization.

## Conclusions

We have identified 67 carotenoid biosynthetic genes in the genome of *B. rapa* through a comparative analysis between *A. thaliana* and *B. rapa*, and established the carotenoid biosynthetic pathway in *B. rapa*. Multiple copies of carotenoid biosynthetic genes were generated and retained after the WGT event and most of them maintained their syntenic relationships with their orthologs in *A. thaliana*. Fisher’s t-test indicated that the proportion of carotenoid biosynthetic genes in each subgenome of *B. rapa* was not significantly different from the whole genome backgrounds. The composition of carotenoid biosynthetic genes could explain the metabolic profiles of carotenoid accumulation and help to elaborate the genetic mechanism of carotenoid biosynthesis in *B. rapa*. The expression analysis of carotenoid biosynthetic genes showed that the paralogs of these genes were differentially expressed among roots, stems, leaves, flowers, calli, and siliques, suggesting that the functional differentiation of the duplicated carotenoid biosynthetic genes occurred after polyploidization.

Our study of the complete set of carotenoid biosynthetic genes in *B. rapa* will provide insights into carotenoid metabolic mechanisms in *Brassica* crops. In addition, a better understanding of carotenoid biosynthetic genes in *B. rapa* will facilitate the development of conventional and transgenic *B. rapa* cultivars with enriched carotenoid levels in the future.

## Methods

### Database for carotenoid biosynthetic gene identification in *B. rapa*

Gene sequences of *A. thaliana* involved in the carotenoid biosynthetic pathway were acquired from the KEGG pathway database (http://www.genome.jp/kegg/pathway.html) and TAIR database (www.arabidopsis.org). The *B. rapa* genome (version 1.5) and a set of annotated gene sequences from BRAD (http://Brassicadb.org) were used to identify the carotenoid biosynthetic genes in *B. rapa* [41]*.*

### Identification and analysis of orthologs between *B. rapa* and *A. thaliana*

The carotenoid biosynthetic gene and protein sequences of *A. thaliana* were aligned with the genome and protein sequences of *B. rapa* using BLASTN and BLASTP, respectively, with a cut off E-value ≤ 1E^−10^ and coverage ≥ 0.75. Syntenic orthologs between *A. thaliana* and *B. rapa* from BRAD (http://brassicadb.org/brad/) were identified based on sequence similarity (cutoff: E ≤ 10^−20^) and the collinearity of flanking genes [63].

### Phylogenetic analysis

The phylogenetic tree was constructed based on the full-length sequences of *PSY* proteins. A neighbor-joining tree was built using MEGA version 5.05 [64], adopting the Poisson correction distance. Support for the tree obtained was assessed using the bootstrap method with 1000 replicates.

### Accession numbers

Sequence data for the article can be found in the TAIR, NCBI, and *Brassica* (BRAD; http://brassicadb.org/brad/) databases. The accession numbers for the sequences are as follows: SlPSY1: NP_001234812.1; SlPSY2: NP_001234671.1; ZmPSY1: ACY70935.1; ZmPSY2: NP_001108117.1; ZmPSY3: NP_001108125.1; HcPSY: AEH05575.1; TaPSY: ABS83386.1; CaPSY: ADH04284.1; OsPSY1: AAS18307.1; OsPSY3: ABC75828.1; BnaC.PSY.a: JF920037; BnaA.PSY.b: JF920038; BnaA.PSY.c: JF920039, and all others can be found in *Brassica* databases.

### Expression analysis of *B. rapa* carotenoid biosynthetic genes

The expression patterns of carotenoid biosynthetic genes in *B. rapa* were measured using RNA-Seq data [53]. Six tissues, root, stem, leaf, flower, callus, and silique, of *B. rapa* accession Chiifu-401-42 were prepared for mRNA extraction. Plants were grown under greenhouse conditions at 22 °C. Callus tissue was obtained from tissue culture. Root, stem, and leaf tissues were collected from 7-week-old plants. Two samples of root and leaf tissues were generated from different batches of plants. Flower tissue was obtained from blooming plants on the same day and excluded the floral shoot. Silique tissue was collected from plants 15 days after pollination [53]. FPKM values are available in Additional file 2: Table S2. Gene expression FPKM values were log2 transformed, and R software was employed to normalize the expression data and calculate hierarchical clustering [54].

### Availability of supporting data

The *B. rapa* genome sequence (version 1.5) and gene sequences were acquired from BRAD (http://brassicadb.org/brad/).

## Additional files

Additional file 1: Table S1. Gene inventory of the carotenoid pathway and the *Brassica rapa* orthologs [65]. Additional file 2: Table S2. FPKM values for carotenoid biosynthetic gene expression levels in *Brassica rapa.*
Additional file 3: Figure S1. Average expression levels of carotenoid biosynthetic genes in three subgenomes of *Brassica rapa*.
