# Supplementary material for: Carotenoid biosynthetic genes in Brassica rapa: comparative genomic analysis, phylogenetic analysis, and expression profiling
Source: BMC Genomics. 2015 Jul 3;16(1):492. doi: 10.1186/s12864-015-1655-5 (PMC4490644; doi:10.1186/s12864-015-1655-5)
Supplement: Additional file 1: Table S1. — Gene inventory of the carotenoid pathway and the Brassica rapa orthologs [65]. [file 12864_2015_1655_MOESM1_ESM.docx]

**Additional file 1: Table S1. Gene inventory of the carotenoids pathway and the *Brassica rapa* orthologs.**

| Name | AGI No. | Br Name | BrID^a^ | Blocks^b^ | Subgenome | References |
| --- | --- | --- | --- | --- | --- | --- |
| MEP pathway to GGPP | | | | | | |
| *DXS* | *AT4G15560* | *BrDXS1* | *Bra033495*(s) | T(A01) | LF | [5] |
|  |  | *BrDXS2* | *Bra012779*(s) | T(A03) | MF1 |  |
| *DXR* | *AT5G62790* | *BrDXR1* | *Bra010123*(s) | X(A06) | LF | [4] |
|  |  | *BrDXR2* | *Bra035881*(s) | X(A09) | MF2 |  |
| *MCT* | *AT2G02500* | *BrMCT* | *Bra026591*(s) | K(A02) | MF1 | [11] |
| *CMK* | *AT2G26930* | *BrCMK* | *Bra012040*(s) | I(A07) | LF | [7] |
| *MDS* | *AT1G63970* | *BrMDS1* | *Bra027672*(s) | D(A09) | MF2 | [8] |
|  |  | *BrMDS2* | *Bra027770* | —(A09) | MF2 |  |
| *HDS* | *AT5G60600* | *BrHDS* | *Bra002468*(s) | Wb(A10) | LF | [6] |
| *HDR* | *AT4G34350* | *BrHDR1* | *Bra011522*(s) | U(A01) | LF | [2] |
|  |  | *BrHDR2* | *Bra034620*(s) | U(A08) | MF2 |  |
| *IPPI1* | *AT5G16440* | *BrIPPI1* | *Bra006354*(s) | R(A03) | MF1 | [3] |
| *IPPI2* | *AT3G02780* | *BrIPPI2.1* | *Bra040599*(s) | F(A05) | LF | [3] |
|  |  | *BrIPPI2.2* | *Bra021411*(s) | F(A01) | MF1 |  |
|  |  | *BrIPPI2.3* | *Bra001063*(s) | F(A03) | MF2 |  |
| *GGPS1* | [*AT4G36810*](http://www.kegg.jp/dbget-bin/www_bget?ath:AT4G36810) | *BrGGPS1.1* | *Bra011709*(s) | U(A01) | LF | [10,13] |
|  |  | *BrGGPS1.2* | *Bra017785*(s) | U(A03) | MF1 |  |
|  |  | *BrGGPS1.3* | *Bra010576*(s) | U(A08) | MF2 |  |
|  |  | *BrGGPS1.4* | *Bra028096* | —(A07) | LF |  |
| *GGPS2* (*GGPS5*) | [*AT2G23800*](http://www.kegg.jp/dbget-bin/www_bget?ath:AT2G23800) | *Br GGPS2.1* | *Bra039216*(s) | I(A09) | LF | [10,12] |
|  |  | *Br GGPS2.2* | *Bra032140*(s) | I(A04) | MF1 |  |
| *GGPS3* | [*AT3G14550*](http://www.kegg.jp/dbget-bin/www_bget?ath:AT3G14550) | *BrGGPS3.1* | *Bra027330*(s) | F(A05) | LF | [10] |
|  |  | *BrGGPS3.2* | *Bra021562*(s) | F(A01) | MF1 |  |
|  |  | *BrGGPS3.3* | *Bra001556*(s) | F(A03) | MF2 |  |
| *GGPS4* | [*AT2G18640*](http://www.kegg.jp/dbget-bin/www_bget?ath:AT2G18640) | *BrGGPS4* | *Bra038544*(s) | H(A09) | MF1 | [10,12] |
| *GGPS7* | [*AT2G18620*](http://www.kegg.jp/dbget-bin/www_bget?ath:AT2G18620) |  |  |  |  | [9] |
| *GGPS6* | [*AT1G49530*](http://www.kegg.jp/dbget-bin/www_bget?ath:AT1G49530) |  |  |  |  | [10,12] |
| *GGPS8* | [*AT3G14510*](http://www.kegg.jp/dbget-bin/www_bget?ath:AT3G14510) | *BrGGPS8.1* | *Bra021565*(s) | F(A01) | MF1 | [9] |
| *GGPS9* | [*AT3G14530*](http://www.kegg.jp/dbget-bin/www_bget?ath:AT3G14530) |  |  |  |  | [9] |
| *GGPS10* | [*AT3G20160*](http://www.kegg.jp/dbget-bin/www_bget?ath:AT3G20160) | *BrGGPS10.1* | *Bra035808*(s) | F(A05) | LF | [9,12] |
|  |  | *BrGGPS10.2* | *Bra001777*(s) | F(A03) | MF2 |  |
| *GGPS11* | [*AT3G29430*](http://www.kegg.jp/dbget-bin/www_bget?ath:AT3G29430) |  |  |  |  | [9] |
| *GGPS12* | [*AT3G32040*](http://www.kegg.jp/dbget-bin/www_bget?ath:AT3G32040) |  |  |  |  | [9] |
| *GGR* | [*AT4G38460*](http://www.kegg.jp/dbget-bin/www_bget?ath:AT4G38460) | *BrGGR* | *Bra011898*(s) | U(A01) | LF | [9] |
| Carotenoid biosynthesis | | | | | | |
| *PSY* | *AT5G17230* | *BrPSY1* | *Bra008569*(s) | R(A10) | LF | [13,14] |
|  |  | *BrPSY2* | *Bra006391*(s) | R(A03) | MF1 |  |
|  |  | *BrPSY3* | *Bra023603*(s) | R(A02) | MF2 |  |
| *PDS3* | *AT4G14210* | *BrPDS3.1* | *Bra032770*(s) | T(A04) | MF1 | [18] |
|  |  | *BrPDS3.2* | *Bra010751*(s) | T(A08) | MF2 |  |
| *Z-ISO* | *AT1G10830* | *BrZ-ISO* | *Bra019899*(s) | A(A06) | LF | [15] |
| *ZDS* | *AT3G04870* | *BrZDS* | *Bra040411*(s) | F(Scaffold000203) | MF1 | [17] |
| *CRTISO* | *AT1G06820* | *BrCRTISO* | *Bra031539*(s) | A(A09) | MF2 | [16] |
| *CRTISO2* | *AT1G57770* | *BrCRTISO2* | *Bra027908*(s) | D(A09) | MF2 | [9] |
| *LYC* | *AT3G10230* | *BrLYC* | *Bra029825*(s) | F(A05) | LF | [19] |
| *LUT2* | *AT5G57030* | *BrLUT2.1* | *Bra002769*(s) | Wb(A10) | LF | [19] |
|  |  | *BrLUT2.2* | *Bra006838*(s) | Wb(A03) | MF1 |  |
|  |  | *BrLUT2.3* | *Bra020718* | —(A02) | MF1 |  |
| *CHY1* | *AT4G25700* | *BrCHY1.1* | *Bra013912*(s) | U(A01) | LF | [22] |
|  |  | *BrCHY1.2* | *Bra019145*(s) | U(A03) | MF1 |  |
| *CHY2* | *AT5G52570* | *BrCHY2* | *Bra003121*(s) | Wb(A10) | LF | [22] |
| *LUT5* | *AT1G31800* | *BrLUT5* | *Bra038437*(s) | B(A08) | MF1 | [21] |
| *CYP97B3* | *AT4G15110* | *Br CYP97B3* | *Bra038092*(s) | T(A08) | MF2 | [23] |
| *LUT1* | *AT3G53130* |  |  |  |  | [22] |
| *ZEP* | *AT5G67030* | *BrZEP1* | *Bra012127*(s) | X(A07) | LF | [24] |
|  |  | *BrZEP2* | *Bra037130*(s) | X(A09) | MF2 |  |
| *VDE* | *AT1G08550* | *BrVDE* | *Bra018616*(s) | A(A06) | LF | [25] |
| *NSY* | *AT1G67080* | *BrNSY* | *Bra034026*(s) | E(A02) | MF1 | [24] |
| *CCD7* | *AT2G44990* | *BrCCD7* | *Bra040330*(s) | J(A04) | MF1 | [20] |
| *CCD8* | *AT4G32810* | *BrCCD8* | *Bra011384*(s) | U(A01) | LF | [20] |
| *NCED2* | [*AT4G18350*](http://www.kegg.jp/dbget-bin/www_bget?ath:AT4G18350) | *BrNCED2.1* | *Bra013298*(s) | U(A01) | LF | [31] |
|  |  | *BrNCED2.2* | *Bra012603*(s) | U(A03) | MF1 |  |
| *NCED3* | [*AT3G14440*](http://www.kegg.jp/dbget-bin/www_bget?ath:AT3G14440) | *BrNCED3.1* | *Bra027336*(s) | F(A05) | LF | [27] |
|  |  | *BrNCED3.2* | *Bra021558*(s) | F(A01) | MF1 |  |
|  |  | *BrNCED3.3* | *Bra001552*(s) | F(A03) | MF2 |  |
| *NCED4* | [*AT4G19170*](http://www.kegg.jp/dbget-bin/www_bget?ath:AT4G19170) | *BrNCED4.1* | *Bra013378*(s) | U(A01) | LF | [26] |
|  |  | *BrNCED4.2* | *Bra020970*(s) | U(A08) | MF2 |  |
| *NCED5* | *AT1G30100* | *BrNCED5* | *Bra032359*(s) | B(A09) | LF | [31] |
| *NCED6* | [*AT3G24220*](http://www.kegg.jp/dbget-bin/www_bget?ath:AT3G24220) | *BrNCED6* | *Bra015002*(s) | F(A07) | LF | [28,31] |
| *NCED9* | [*AT1G78390*](http://www.kegg.jp/dbget-bin/www_bget?ath:AT1G78390) | *BrNCED9.1* | *Bra035033*(s) | E(A07) | LF | [28] |
|  |  | *BrNCED9.2* | *Bra008358*(s) | E(A02) | MF1 |  |
| *ABA2* | *AT1G52340* | *BrABA2.1* | *Bra018964*(s) | C(A06) | LF | [29] |
|  |  | *BrABA2.2* | *Bra014323*(s) | C(A08) | MF1 |  |
| *AAO3* | *AT2G27150* | *BrAAO3* | *Bra034325*(s) | I(A04) | MF1 | [30] |

^a^ “(s)”indicates carotenoid biosynthetic genes that exhibit synteny.

^b^ Characters referring to 24 conserved blocks (A–X) that represent conserved segments identifiable in the ancestral karyotype, *A. thaliana* and *B. rapa* [65]. Two letters indicate that the gene is located at the boundary between two blocks; text in parentheses indicates the specific chromosome of *B. rapa*. Bra040411 was anchored on Scaffold000203, which has not yet been mapped onto a chromosome according to the new genome version.
