# Supplementary material for: Carotenoid biosynthetic genes in Brassica rapa: comparative genomic analysis, phylogenetic analysis, and expression profiling
Source: BMC Genomics. 2015 Jul 3;16(1):492. doi: 10.1186/s12864-015-1655-5 (PMC4490644; doi:10.1186/s12864-015-1655-5)
Supplement: Additional file 2: Table S2. — FPKM values for carotenoid biosynthetic gene expression levels in Brassica rapa. [file 12864_2015_1655_MOESM2_ESM.docx]

**Additional file 2: Table S2. FPKM values for the carotenoid biosynthetic gene expression in *Brassica rapa.***

| Br Name | BrID | FPKM | | | | | | | |
| --- | --- | --- | --- | --- | --- | --- | --- | --- | --- |
|  |  | Root_1 | Root_2 | Stem | Leaf_1 | Leaf_2 | Flower | Silique | Callus |
| *BrDXS1* | Bra033495 | 16.0432 | 17.2068 | 28.5492 | 29.987 | 33.2411 | 39.9453 | 12.4711 | 25.2245 |
| *BrDXS2* | Bra012779 | 6.58891 | 7.60031 | 14.2733 | 29.9701 | 29.3934 | 19.1892 | 15.9545 | 20.6056 |
| *BrDXR1* | Bra010123 | 12.4607 | 11.1392 | 11.362 | 36.965 | 28.0202 | 23.412 | 18.4667 | 10.2615 |
| *BrDXR2* | Bra035881 | 3.05951 | 1.64205 | 5.76651 | 11.1909 | 8.67931 | 6.12085 | 6.70646 | 1.48709 |
| *BrMCT* | Bra026591 | 6.05414 | 2.10839 | 7.96432 | 21.3745 | 14.8948 | 6.43123 | 7.76286 | 2.0232 |
| *BrCMK* | Bra012040 | 3.76583 | 3.58707 | 7.35405 | 13.3957 | 12.5182 | 5.46098 | 4.85238 | 4.22699 |
| *BrMDS1* | Bra027672 | 5.01311 | 6.6079 | 10.7659 | 41.679 | 30.5019 | 20.036 | 19.784 | 15.9126 |
| *BrMDS2* | Bra027770 | 5.01311 | 4.78834 | 5.33533 | 4.58668 | 7.60013 | 5.6412 | 4.946 | 7.53756 |
| *BrHDS* | Bra002468 | 18.928 | 20.4351 | 52.3986 | 95.2815 | 103.898 | 254.419 | 53.788 | 44.2858 |
| *BrHDR1* | Bra011522 | 5.15306 | 4.66871 | 4.37389 | 25.0858 | 18.804 | 128.126 | 1.67079 | 7.76581 |
| *BrHDR2* | Bra034620 | 23.4337 | 20.0296 | 35.117 | 64.415 | 54.6079 | 77.4626 | 76.6694 | 43.6187 |
| *BrIPPI1* | Bra006354 | 1.49418 | 2.67549 | 0.565812 | 1.37463 | 0.771919 | 2.68291 | 1.42218 | 1.62275 |
| *BrIPPI2.1* | Bra040599 | 71.3175 | 82.1789 | 56.3305 | 47.2023 | 58.9236 | 43.1046 | 138.322 | 71.0724 |
| *BrIPPI2.2* | Bra021411 | 2.53578 | 2.91068 | 0.386022 | 0.40347 | 0.410664 | 0.787097 | 1.3897 | 1.48271 |
| *BrIPPI2.3* | Bra001063 | 51.35 | 55.9706 | 50.1048 | 27.2091 | 38.1805 | 13.89 | 35.3994 | 21.1607 |
| *BrGGPS1.1* | Bra011709 | 16.3133 | 29.2296 | 57.0719 | 42.3849 | 63.4087 | 107.571 | 67.9291 | 30.414 |
| *BrGGPS1.2* | Bra017785 | 0.787065 | 0.628966 | 3.48471 | 0.801821 | 0.671443 | 3.09929 | 0 | 7.67108 |
| *BrGGPS1.3* | Bra010576 | 0.109146 | 0.102935 | 0 | 0 | 0 | 0.110501 | 0 | 1.42282 |
| *BrGGPS1.4* | Bra028096 | 8.71235 | 14.6649 | 10.2451 | 13.3131 | 18.2399 | 8.02811 | 7.36314 | 3.51881 |
| *BrGGPS2.1* | Bra039216 | 0 | 0.112737 | 0 | 0 | 0 | 0.721596 | 0.510659 | 0.516371 |
| *BrGGPS2.2* | Bra032140 | 0.163786 | 1.17769 | 0.114342 | 0 | 0 | 0.083005 | 0.352797 | 0.088974 |
| *BrGGPS3.1* | Bra027330 | 0 | 0.105881 | 0 | 0 | 0 | 0 | 0 | 0.121745 |
| *BrGGPS3.2* | Bra021562 | 0 | 0 | 0 | 0 | 0 | 0 | 0 | 0 |
| *BrGGPS3.3* | Bra001556 | 0 | 0 | 0 | 0 | 0 | 0 | 0.321201 | 0 |
| *BrGGPS4* | Bra038544 | 0.435164 | 0.410311 | 0 | 0 | 0.162694 | 0 | 0 | 0.35455 |
| *BrGGPS8.1* | Bra021565 | 0 | 0 | 0 | 0 | 0 | 0 | 0 | 0 |
| *BrGGPS10.1* | Bra035808 | 0.690944 | 1.0355 | 0 | 0 | 0 | 0 | 0 | 0.750573 |
| *BrGGPS10.2* | Bra001777 | 0.339626 | 1.33787 | 0 | 0 | 0 | 0 | 0 | 0 |
| *BrGGR* | Bra011898 | 18.8612 | 26.7373 | 56.2052 | 33.2716 | 51.6673 | 39.5798 | 50.8986 | 29.0705 |
| *BrPSY1* | Bra008569 | 2.10513 | 1.87899 | 8.30594 | 34.5477 | 23.5221 | 33.8474 | 27.0574 | 5.56849 |
| *BrPSY2* | Bra006391 | 1.88735 | 0.926191 | 3.80195 | 11.7419 | 8.90617 | 140.435 | 6.63439 | 5.33112 |
| *BrPSY3* | Bra023603 | 4.31655 | 2.54289 | 5.58145 | 18.6819 | 14.6474 | 7.12984 | 13.3295 | 2.95645 |
| *BrPDS3.1* | Bra032770 | 0 | 0 | 0.030675 | 0.137885 | 0.12913 | 0.202163 | 8.5006 | 0 |
| *BrPDS3.2* | Bra010751 | 8.53986 | 9.80299 | 17.2679 | 42.0729 | 36.5446 | 49.7845 | 12.179 | 5.69887 |
| *BrZ-ISO* | Bra019899 | 3.49764 | 4.85229 | 7.20578 | 16.2539 | 23.6244 | 36.7758 | 16.8142 | 4.90262 |
| *BrZDS* | Bra040411 | 26.9495 | 31.6493 | 25.2569 | 67.279 | 67.7178 | 133.078 | 39.4114 | 12.4665 |
| *BrCRTISO* | Bra031539 | 6.44428 | 6.89864 | 9.14334 | 11.8013 | 11.3053 | 7.6264 | 6.26816 | 3.36376 |
| *BrCRTISO2* | Bra027908 | 0.509281 | 0.438222 | 2.46522 | 29.3972 | 27.0197 | 4.26188 | 3.75278 | 1.24507 |
| *BrLYC* | Bra029825 | 5.40193 | 6.60428 | 10.0317 | 23.4555 | 25.8878 | 42.7156 | 15.076 | 6.20453 |
| *BrLUT2.1* | Bra002769 | 0 | 0 | 1.51366 | 4.49992 | 1.83609 | 1.6583 | 3.7806 | 1.85409 |
| *BrLUT2.2* | Bra006838 | 0 | 0 | 5.79873 | 20.3477 | 22.2463 | 14.7181 | 3.96817 | 1.69239 |
| *BrLUT2.3* | Bra020718 | 0 | 0 | 0 | 0 | 0 | 0.162832 | 0 | 0 |
| *BrCHY1.1* | Bra013912 | 3.38207 | 3.19348 | 4.88906 | 12.3335 | 12.085 | 13.8232 | 16.607 | 4.47197 |
| *BrCHY1.2* | Bra019145 | 34.2181 | 21.5769 | 4.00429 | 22.7622 | 14.4837 | 81.4358 | 55.3111 | 15.1086 |
| *BrCHY2.1* | Bra003121 | 0 | 0 | 0 | 0 | 0 | 3.1953 | 0.786131 | 0 |
| *BrLUT5* | Bra038437 | 2.20951 | 2.76554 | 8.96969 | 49.5759 | 41.4298 | 67.6417 | 7.443 | 8.10239 |
| *BrCYP97B3* | Bra038092 | 8.38812 | 7.55799 | 15.2128 | 58.0491 | 37.4738 | 24.3842 | 14.4323 | 4.95175 |
| *BrZEP1* | Bra012127 | 8.46645 | 9.41309 | 42.3883 | 210.449 | 255.566 | 129.09 | 26.101 | 35.2455 |
| *BrZEP2* | Bra037130 | 3.22012 | 3.04046 | 2.67919 | 2.06397 | 2.12942 | 70.7208 | 12.0806 | 4.23366 |
| *BrVDE* | Bra018616 | 0.168319 | 0.586411 | 10.2744 | 42.5089 | 54.8431 | 6.82351 | 2.41668 | 6.12618 |
| *BrNSY* | Bra034026 | 33.3479 | 23.7996 | 21.238 | 42.0651 | 29.1995 | 32.986 | 71.0548 | 12.5255 |
| *BrNCED2.1* | Bra013298 | 0 | 0.088342 | 0 | 0 | 0 | 1.04109 | 1.93684 | 0.069701 |
| *BrNCED2.2* | Bra012603 | 0 | 0.28495 | 0.204119 | 0 | 0 | 0.443132 | 0.87881 | 0.285059 |
| *BrNCED3.1* | Bra027336 | 11.3897 | 17.2374 | 5.10353 | 3.22943 | 4.85652 | 8.27082 | 5.01181 | 1.35254 |
| *BrNCED3.2* | Bra021558 | 8.26241 | 14.6407 | 5.1796 | 4.31942 | 8.06858 | 3.59209 | 15.1863 | 0.337506 |
| *BrNCED3.3* | Bra001552 | 0.198831 | 0.419441 | 0.466978 | 0.61842 | 0.640569 | 3.72557 | 1.85365 | 0 |
| *BrNCED4.1* | Bra013378 | 1.731 | 1.0085 | 4.29343 | 18.1917 | 22.4611 | 24.5698 | 8.21743 | 7.52292 |
| *BrNCED4.2* | Bra020970 | 0.197754 | 0.272621 | 0.243395 | 0.1368 | 0.096054 | 2.60746 | 0 | 0.358116 |
| *BrNCED5* | Bra032359 | 0.189896 | 0.261385 | 0.291758 | 0.131373 | 0.368374 | 0.128417 | 0.182028 | 0.137556 |
| *BrNCED6* | Bra015002 | 0.06366 | 0.087644 | 0 | 0 | 0 | 0.129148 | 5.12575 | 0 |
| *BrNCED9.1* | Bra035033 | 2.09662 | 2.65702 | 0.454381 | 0.127989 | 0.209135 | 1.43878 | 93.5524 | 0.067004 |
| *BrNCED9.2* | Bra008358 | 0 | 0 | 0 | 0 | 0.030758 | 0 | 0.091186 | 0 |
| *BrABA2.1* | Bra018964 | 42.4682 | 60.9858 | 31.4113 | 24.8411 | 35.2616 | 19.1275 | 11.0758 | 19.0978 |
| *BrABA2.2* | Bra014323 | 0.171776 | 0.079876 | 0.120171 | 0.356305 | 0.422216 | 0.783354 | 0.493205 | 0.093313 |
| *BrAAO3* | Bra034325 | 2.84462 | 3.15516 | 2.3945 | 2.8324 | 2.55198 | 4.35245 | 2.97454 | 1.73616 |
| *BrCCD7* | Bra040330 | 0.824654 | 0.828352 | 0.176618 | 0 | 0 | 0.055786 | 0 | 0 |
| *BrCCD8* | Bra011384 | 13.684 | 21.4326 | 10.6876 | 0.068266 | 0.12781 | 1.40126 | 0.945774 | 0 |
