# Supplementary figures and images for: Carotenoid biosynthetic genes in Brassica rapa: comparative genomic analysis, phylogenetic analysis, and expression profiling
Source: BMC Genomics. 2015 Jul 3;16(1):492. doi: 10.1186/s12864-015-1655-5 (PMC4490644; doi:10.1186/s12864-015-1655-5)

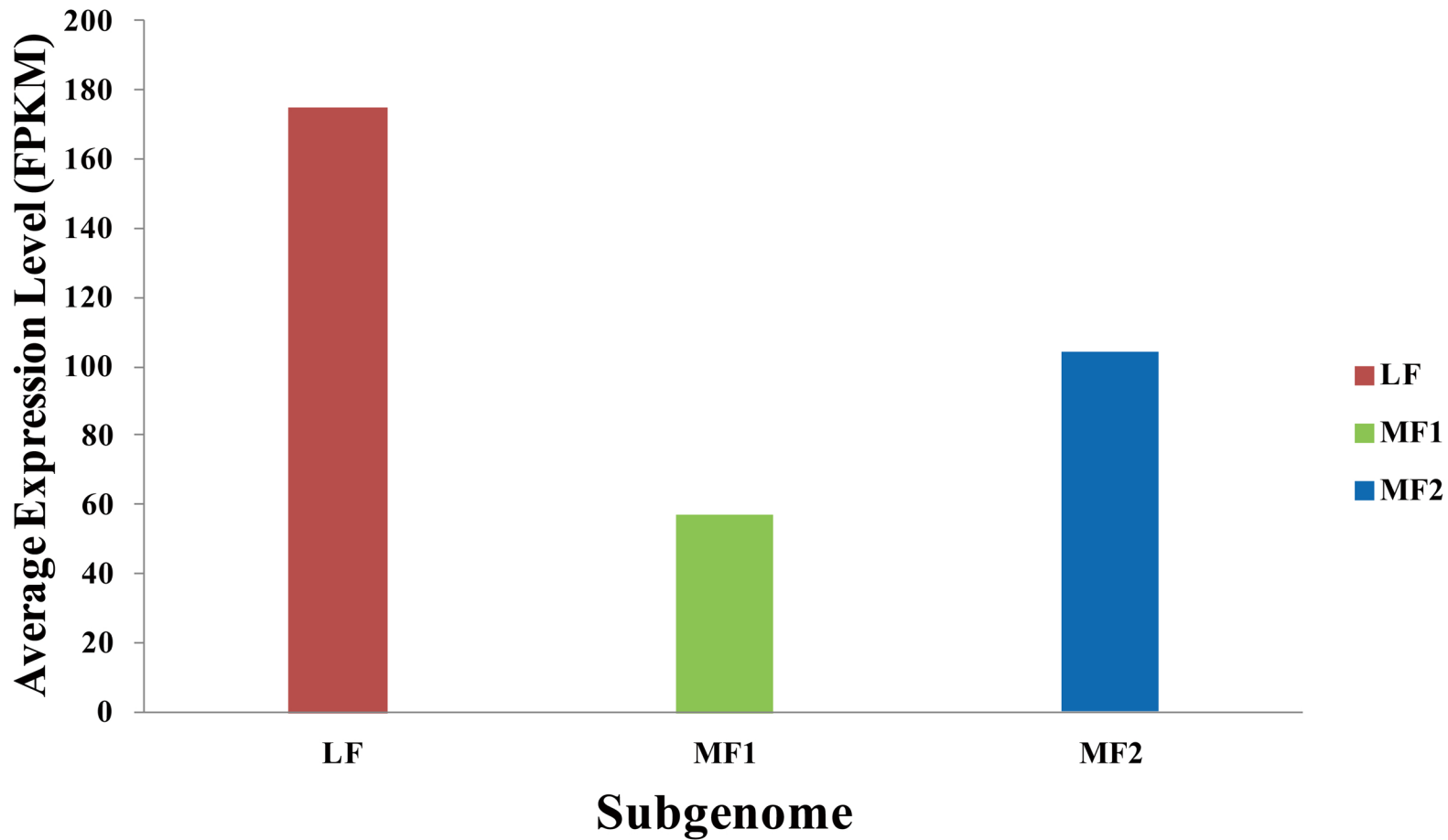

Supplement: Additional file 3: Figure S1. — Average expression levels of carotenoid biosynthetic genes in three subgenomes of Brassica rapa. [file 12864_2015_1655_MOESM3_ESM.pdf]
